# Preliminary investigation on feline coronavirus presence in the reproductive tract of the tom cat as a potential route of viral transmission

**DOI:** 10.1177/1098612X19837114

**Published:** 2019-03-22

**Authors:** Angelica Stranieri, Monica Probo, Maria C Pisu, Alberto Fioletti, Sara Meazzi, Maria E Gelain, Federico Bonsembiante, Stefania Lauzi, Saverio Paltrinieri

**Affiliations:** 1 Department of Veterinary Medicine, University of Milan, Milan, Italy; 2 Central Laboratory, Veterinary Teaching Hospital, University of Milan, Lodi, Italy; 3 Veterinary Reference Centre, Turin, Italy; 4 Department of Comparative Biomedicine and Food Science, University of Padova, Legnaro, Padova, Italy

**Keywords:** Feline coronavirus, feline infectious peritonitis, tom cat reproduction, cattery management, PCR, prevention

## Abstract

**Objectives:**

Feline infectious peritonitis (FIP) is an immune-mediated disease initiated by feline coronavirus (FCoV) infection. To date, the only proven route of transmission is the faecal–oral route, but a possible localisation of FCoV in the reproductive tract of tom cats is of concern, owing to the involvement of the male reproductive tract during FIP and to the presence of reproduction disorders in FCoV-endemic feline catteries. The aim of the study was to investigate the presence and localisation of FCoV in semen and/or in the reproductive tract of tom cats, and its possible association with seroconversion and viraemic phase.

**Methods:**

Blood, serum, semen and/or testicle samples were obtained from 46 tom cats. Serology was performed on 38 serum samples, nested reverse transcriptase PCR (nRT-PCR) and reverse transcriptase quantitative PCR (RT-qPCR) were performed on 39 blood samples and on 17 semen samples, and histology, immunohistochemistry and nRT-PCR were performed on 39 testicles.

**Results:**

Twenty-four of 38 serum samples were positive on serology. Semen samples were negative on RT-PCR and RT-qPCR for FCoV, while all blood samples were negative at both molecular methods, except for one sample positive at RT-qPCR with a very low viral load. All testicles were negative at immunohistochemistry, while six were positive at nRT-PCR for FCoV. Serology and blood PCR results suggest that the virus was present in the environment, stimulating transient seroconversion. FCoV seems not to localise in the semen of tom cats, making the venereal route as a way of transmission unlikely. Although viral RNA was found in some testicles, it could not be correlated with the viraemic phase.

**Conclusions and relevance:**

In the light of these preliminary results, artificial insemination appears safer than natural mating as it eliminates the direct contact between animals, thus diminishing the probability of faecal–oral FCoV transmission.

## Introduction

Feline infectious peritonitis (FIP) is an immune-mediated disease of young cats. The causative agent is feline coronavirus (FCoV), generated by a mutation of the widespread enteric pathotype, that gains the ability to replicate in macrophages, and spreads through infected monocytes.^1^ The course of the infection depends, in part, upon the type and strength of the immune response of the host,^2–4^ but environmental factors, such as the level of stress and overcrowding, also play a role.^5^ FCoV infection is very common in cats; around 40% of the domestic cat population has been infected with FCoV, and this figure may increase up to 90% in multi-cat households.^6,7^ Natural FCoV infections are transient in ~70% of cats, but persistent infections can occur in ~13% of cats,^8^ while around 5–10% of cats are believed to be resistant to FCoV infection. In most cases, FCoV infection is asymptomatic, or results in only mild gastrointestinal clinical signs; however, in a small percentage of cases, FCoV infection results in FIP.^5^

Asymptomatic FCoV infection was previously believed to be confined to the intestinal tract, but it is now known that healthy FCoV-infected cats can have systemic infection, albeit with lower viral loads than cats with FIP.^9^ These recurrent phases of intestinal colonisation and faecal shedding of the virus may lead to a transient localisation in several organs and are followed by seroconversion and negativisation at the intestinal level.^10,11^ During the viraemic phase, it is possible that the virus also localises in the reproductive tract, and that it is shed with semen, contributing to the spread of the FCoV by coupling or by artificial insemination (AI) in breeding cats.

Nowadays, AI has become reasonably successful in the domestic cat, sufficiently so to contribute to genetic management of catteries.^12^ Therefore, there is concern about the possibility of sexual transmission of viruses through AI. It has been demonstrated that feline immunodeficiency virus is shed with semen, and that it can be transmitted horizontally by AI with fresh semen.^13^ Feline leukaemia virus infection alters hormone production in the hypothalamic–pituitary–gonadal system, decreasing testosterone, luteinising hormone and follicle-stimulating hormone levels, but its exact localisation in the reproductive system is still unknown.^14^ The involvement of the male reproductive tract during FCoV infection has previously been described as scrotal swelling following abdominal effusion, orchitis or priapism.^15–18^ In all these cases, cats with FCoV in the male reproductive tract were affected by FIP. Nevertheless, the hypothesis of a possible association between FCoV infection and reproductive disorders is supported also by the presence of hypofertility, abortions and/or natimortality in FCoV-endemic catteries.^1^

To the best of our knowledge, localisation of FCoV in the reproductive tract of healthy cats or its presence in tom cat semen has never been demonstrated, but it could represent an important step in the process of understanding the mechanisms of FCoV transmission as, to date, the only proven route of transmission is the faecal–oral route.^4^ Therefore, the aim of the study was to investigate the presence and localisation of FCoV in semen and/or in the reproductive tract of healthy tom cats and its possible association with seroconversion or with the viraemic phase.

## Material and methods

### Sample collection

Blood, serum, semen and/or testicle samples were obtained from 46 cats aged from 6 months to 4 years. Seven cats were tom cats from breeding catteries and their semen samples were collected for AI purposes. All remaining cats were client-owned, except for two stray cats. One of the stray cats underwent orchiectomy after being placed in a shelter; the other was found severely injured and euthanased.

Blood samples were available if routine haematology and/or biochemistry were performed prior to semen collection and/or surgery. After routine diagnostic procedures performed at the site of collection, blood or serum samples, when available, were immediately frozen and periodically sent to the Laboratory of the Veterinary Teaching Hospital of the University of Milan in cold chain. Semen samples were collected as described below, either for AI purposes or before orchiectomy, with the owner’s informed consent.

Testicle samples were obtained after orchiectomy from all of the cats except two; for these the testicles were collected during necropsy performed for diagnostic purposes. Immediately after collection, half the testicle was frozen in a plain tube, while the other half was placed into 10% neutral-buffered formalin for histological and immunohistochemical examination. For the two cats that had necropsy performed, tissue samples grossly affected by lesions were also collected into 10% neutral-buffered formalin for histology and immunohistochemistry to reach a definitive diagnosis.

The study protocol was approved by the Institutional Animal Care and Use Committee of the University of Milan (approval number 109/2016).

### Semen collection

Semen samples were collected at the Veterinary Reference Centre (Turin, Italy) via urethral catheterisation using an injectable anaesthesia protocol with 0.2 mg/kg methadone (Semfortan; Dechra) and 5 µg/kg dexmedetomidine (Dexdomitor; Pfizer Italia) premedication, followed by induction with 2 mg/kg propofol (Propovet; Esteve Veterinaria) to effect.^19^ Immediately after collection, semen samples were frozen and sent to the laboratory of the Veterinary Teaching Hospital of the University of Milan, maintaining the cold chain for molecular biology processing.

### Serology

Anti-FCoV antibody titres were assessed using an indirect immunofluorescence test performed on 10 multi-well slides produced at the University of Zurich according to Osterhaus et al,^20^ by coating each well with 4.5 × 10³ PD-5 cells, half of which were infected with swine transmissible gastroenteritis virus (serologically cross-reacting with FCoV). Two-fold dilutions (1:25–1:400) of each serum sample were prepared and 20 µl of each dilution was applied to the wells. After incubation for 30 mins at 37°C in a moist chamber, slides were washed with phosphate-buffered saline (PBS), dried and 15 µl of fluorescein isothiocyanate-conjugated rabbit-anti-cat immunoglobulin (Nordic Immunological Laboratories) was added to each well. After incubation for 30 mins at 37°C in a moist chamber, slides were washed, dried and observed on a fluorescence microscope. Dilutions were judged as positive when showing a clear fluorescent signal in about half of the cells. Samples that were still positive at a 1:400 dilution were further diluted on a two-fold basis until negativisation.

### RNA extraction, nested reverse transcriptase PCR and reverse transcriptase quantitative PCR

RNA was obtained from blood and testicle samples using a NucleoSpin RNA kit (Macherey-Nagel). Fifty microlitres of blood were suspended in 300 µl RA1 lysis buffer, while 20 mg of testicle tissue was thinly shredded on sterile plates using sterile scalpels, followed by vigorous vortexing in RA1 lysis buffer until completely dissolved. All subsequent steps were performed according to the manufacturer’s instructions.

RNA was obtained from semen using TRIzol reagent (Invitrogen) according to Das et al.^21^ Samples (starting mean volume 50 µl; range 8–100 µl) were centrifuged (5 mins at 7000 *g*) and the supernatant was discarded. The resulting pellets were washed twice in 100 µl PBS for 5 mins at 7000 *g*. To each sample, a volume of TRIzol (Thermo Fischer Scientific) equal to 10 × the starting volume of semen was added. After incubation for 5 mins, 200 µl of chloroform for each millilitre of TRIzol was added to each sample. After vortexing and incubating at room temperature for 3 mins, samples were centrifuged (15 mins at 12,000 *g* at 4°C) and the resulting aqueous phase was transferred in RNAse free tubes. Then, 500 µl of isopropyl alcohol for every millilitre of TRIzol was added to each sample, followed by 10 mins incubation at room temperature. After centrifugation (10 mins at 12,000 *g* at 4°C), the resulting supernatant was eliminated and to each resulting pellet 1 ml of 75% ethanol was added. After centrifugation (5 mins at 12,000 *g* at 4°C), supernatant was discarded, and the sample was dried for 10–15 mins at room temperature. The pellet was then suspended in 30 µl of RNAse-free water and incubated at 55°C for 10 mins. RNA samples were then frozen at –80°C or immediately used for nested reverse transcriptase PCR (nRT-PCR).

An nRT-PCR targeting a 177 bp product of the highly conserved 3′ untranslated region of the genome of both type I and type II FCoV was used.^10^ nRT-PCR positive FCoV RNA from a cat with FIP was used as a positive control and RNase-free water as a negative control. PCR products were visualised under an ultraviolet transilluminator on a 1.5% agarose gel stained with ethidium bromide.

Quantitative RT-qPCR targeting a 102 bp product of the *7b* gene of FCoV was performed on blood and semen samples as previously described,^22^ with minor modifications. Threshold cycle (C_T_) number was used as the measure of viral load. The lower the C_T_, the more virus is present in the sample.

### Histopathology and immunohistochemistry

Formalin-fixed samples were sent to the department of Comparative Biomedicine and Food Science of the University of Padova for histology and immunohistochemistry (IHC). Sections (3 µm) obtained from paraffin-embedded samples were prepared and stained with haematoxylin and eosin for histology with an automated stainer (Autostainer XL; Leica Biosystems). For IHC, 3 µm paraffin sections were placed on surface-coated slides (Superfrost Plus). Slides were incubated at 37°C for 30 mins before the immunostaining was performed with an automatic immunostainer (Ventana Benchmark XT; Roche-Diagnostics), which uses a kit with a secondary antibody with a horseradish peroxidase-conjugated polymer that binds mouse and rabbit primary antibodies (ultraViews Universal DAB; Ventana Medical System). All reagents were dispensed automatically except for the primary antibody, which was dispensed by hand. A mouse monoclonal antibody against the FCoV was used as primary antibody (clone FIPV3-70; Serotec).

## Results

### Caseload

The caseload included 31 domestic shorthair cats, six Maine Coons, three Sphynxes and one each of the following breeds: Birman, Chartreux, Norwegian Forest Cat, Persian, Ragdoll and Scottish Fold. Age ranged from 6 to 48 months (mean 11.6; median 7.5 months). The type of samples collected in the 46 cats included in this study is summarised in Table 1. Seventeen semen samples were collected: in all these cases additional samples from the same cats were available (serum, blood and testicle in seven cases; serum and blood in three cases; serum in two cases; blood and testicle in two cases; blood in two cases; serum and testicle in one case).

**Table 1:** Data on signalment and type of sample collected from the cats and included in this study

Cat number	Breed	Age (months)	Samples	Total
1	Persian	12	Blood, serum, testicle	24
2	Domestic shorthair	6	Blood, serum, testicle	
3	Domestic shorthair	7	Blood, serum, testicle	
4	Domestic shorthair	6	Blood, serum, testicle	
5	Domestic shorthair	6	Blood, serum, testicle	
6	Domestic shorthair	8	Blood, serum, testicle	
7	Domestic shorthair	6	Blood, serum, testicle	
8	Domestic shorthair	6	Blood, serum, testicle	
9	Domestic shorthair	7	Blood, serum, testicle	
10	Domestic shorthair	6	Blood, serum, testicle	
11	Domestic shorthair	7	Blood, serum, testicle	
12	Domestic shorthair	7	Blood, serum, testicle	
13	Domestic shorthair	8	Blood, serum, testicle	
14	Domestic shorthair	7	Blood, serum, testicle	
15	Domestic shorthair	7	Blood, serum, testicle	
16	Domestic shorthair	9	Blood, serum, testicle	
17	Domestic shorthair	8	Blood, serum, testicle	
18	Domestic shorthair	7	Blood, serum, testicle	
19	Domestic shorthair	24	Blood, serum, testicle	
20	Domestic shorthair	6	Blood, serum, testicle	
21	Domestic shorthair	7	Blood, serum, testicle	
22	Birman	9	Blood, serum, testicle	
23	Domestic shorthair	36	Blood, serum, testicle	
24	Sphynx	11	Blood, serum, testicle	
25	Ragdoll	13	Blood, serum, semen, testicle	7
26	Domestic shorthair	24	Blood, serum, semen, testicle	
27	Sphynx	11	Blood, serum, semen, testicle	
28	Domestic shorthair	6	Blood, serum, semen, testicle	
29	Maine Coon	14	Blood, serum, semen, testicle	
30	Scottish Fold	11	Blood, serum, semen, testicle	
31	Domestic shorthair	7	Blood, serum, semen, testicle	
32	Maine Coon	27	Blood, serum, semen	3
33	Chartreux	9	Blood, serum, semen	
34	Maine Coon	48	Blood, serum, semen	
35	Norwegian Forest Cat	12	Serum, semen	2
36	Sphynx	10	Serum, semen	
37	Maine Coon	18	Blood, semen	2
38	Maine Coon	30	Blood, semen	
39	Maine Coon	25	Blood, semen, testicle	2
40	Domestic shorthair	7	Blood, semen, testicle	
41	Domestic shorthair	6	Testicle	3
42	Domestic shorthair	7	Testicle	
43	Domestic shorthair	6	Testicle	
44	Domestic shorthair	8	Serum, testicle	1
45	Domestic shorthair	7	Serum, semen, testicle	1
46	Domestic shorthair	6	Blood, testicle	1

A total of 39 testicles were collected, 24 of which were collected along with blood and serum samples. The remaining testicles were collected along with blood, serum and semen (seven cats), with blood and semen (two cats), alone (three cats), with serum (one cat), with serum and semen (one cat), and with blood only (one cat).

### Serology, PCR and IHC

Results obtained for each test are shown in Table 2. Fourteen of the 38 cats for which serum was available were negative on serology, with an antibody titre lower than the cut-off of 1:50, which is the threshold of positivity of our laboratory, while 7/38 cats showed an antibody titre of 1:50. The remaining 17 cats showed variable antibody titres; specifically, the antibody titre was 1:100 in seven cats, 1:200 in six cats, 1:400 in three cats and 1:800 in one cat.

**Table 2:** Results of the test performed on each cat involved in the study

		nRT-PCR			RT-qPCR		
Cat number	Serology	Blood	Semen	Testicle	Blood	Semen	IHC
4	1:800	Neg	NA	Neg	Neg	NA	Neg
8	1:400	Neg	NA	Neg	Neg	NA	Neg
9	1:400	Neg	NA	Neg	Neg	NA	Neg
15	1:200	Neg	NA	Pos	Neg	NA	Neg
6	1:200	Neg	NA	Neg	Neg	NA	Neg
17	1:200	Neg	NA	Neg	Neg	NA	Neg
22	1:200	Neg	NA	Neg	Neg	NA	Neg
24	1:200	Neg	NA	Neg	Neg	NA	Neg
2	1:100	Neg	NA	Neg	Neg	NA	Neg
10	1:100	Neg	NA	Neg	Neg	NA	Neg
12	1:100	Neg	NA	Neg	Neg	NA	Neg
18	1:100	Neg	NA	Pos	Neg	NA	Neg
21	1:100	Neg	NA	Neg	Neg	NA	Neg
14	1:50	Neg	NA	Neg	Neg	NA	Neg
20	1:50	Neg	NA	Neg	Neg	NA	Neg
23	1:50	Neg	NA	Neg	Neg	NA	Neg
1	1:25	Neg	NA	Neg	Pos	NA	Neg
3	<1:25	Neg	NA	Neg	Neg	NA	Neg
5	<1:25	Neg	NA	Neg	Neg	NA	Neg
7	<1:25	Neg	NA	Neg	Neg	NA	Neg
11	<1:25	Neg	NA	Neg	Neg	NA	Neg
13	<1:25	Neg	NA	Neg	Neg	NA	Neg
16	<1:25	Neg	NA	Neg	Neg	NA	Neg
19	<1:25	Neg	NA	Pos	Neg	NA	Neg
29	1:400	Neg	Neg	Pos	Neg	Neg	Neg
27	1:100	Neg	Neg	Neg	Neg	Neg	Neg
25	1:50	Neg	Neg	Neg	Neg	Neg	Neg
26	1:50	Neg	Neg	Neg	Neg	Neg	Neg
28	1:50	Neg	Neg	Neg	Neg	Neg	Neg
30	1:50	Neg	Neg	Neg	Neg	Neg	Neg
31	<1:50	Neg	Neg	Neg	Neg	Neg	Neg
32	<1:25	Neg	Neg	NA	Neg	Neg	NA
33	<1:25	Neg	Neg	NA	Neg	Neg	NA
34	<1:25	Neg	Neg	NA	Neg	Neg	NA
36	1:200	NA	Neg	NA	NA	Neg	NA
35	<1:25	NA	Neg	NA	NA	Neg	NA
44	1:200	NA	NA	Neg	NA	NA	Neg
45	<1:50	NA	Neg	Neg	NA	Neg	Neg
37	NA	Neg	Neg	NA	Neg	Neg	NA
38	NA	Neg	Neg	NA	Neg	Neg	NA
39	NA	Neg	Neg	Neg	Neg	Neg	Neg
40	NA	Neg	Neg	Neg	Neg	Neg	Neg
41	NA	NA	NA	Neg	NA	NA	Neg
42	NA	NA	NA	Pos	NA	NA	Neg
43	NA	NA	NA	Pos	NA	NA	Neg
46	NA	Neg	NA	Neg	Neg	NA	Neg

nRT-PCR = nested reverse transcriptase PCR; RT-qPCR = reverse transcriptase quantitative PCR; IHC = immunohistochemistry for feline coronavirus; Neg = negative; NA = specimen not available; Pos = positive

All 17 semen samples were negative at both the nRT-PCR and the RT-qPCR for FCoV. All 39 blood samples were negative at the nRT-PCR and at the RT-qPCR, except for one blood sample that was FCoV positive only using RT-qPCR, with a very high C_T_ value (C_T_ 38.9).

Regarding testicles, all the cats were negative at immunohistochemistry for FCoV, while six were positive in nRT-PCR for FCoV. All the cats from which testicles were collected while alive were healthy during orchiectomy, except for one cat (cat 43), which was affected by a congenital portosystemic shunt. For two cats (cats 42 and 43) serum and blood were not available and therefore serology was not performed. Antibody titres of the remaining cats with PCR-positive testicles were negative (cat 5); 1:100 (cat 18); 1:200 (cat 15) and 1:400 (cat 29). Interestingly, the only cat affected by FIP, as confirmed by positive immunohistochemistry for FCoV on brain and cerebellum, gave a negative result both with immunohistochemistry and PCR on testicles.

## Discussion

FCoV RNA was never detected by nRT-PCR in the blood samples obtained from the cats examined in this study and only 1/39 blood samples was identified as positive by RT-qPCR. The very high C_T_ value of the positive sample suggests that the concentration of viral RNA in the sample was extremely low. The RT-qPCR positivity resulted in a seronegative cat and this is in accordance with FCoV infection kinetics.^23^ Antibody titres were variable, with mostly medium-to-low titres, while titres >1:200 were found only in a few cases. Taken together, the serology and blood PCR results suggested that the virus was present in the environment and stimulated transient seroconversion in some of the cats.

Positive serology in cats without viral RNA in blood is, in fact, unlikely to be imputable to a low viral load in blood because samples were analysed by RT-qPCR, which is a very sensitive method, and it is more likely that the results are due to the characteristics of FCoV–host interactions.^4,10,24^ It is also possible that an infected cat could not be identified with PCR on blood if the virus was present in the intestinal tract only. Unfortunately, our study design did not include faecal sampling and it is therefore impossible to confirm that seropositive and PCR-negative cats were shedding the virus with faeces. However, positive serology demonstrates that the cats included in this study had been in contact with the virus, as cats may remain positive also after the clearance of the virus. In particular, antibodies against FCoV are typically fluctuating and cats, especially those from multi-cat environments, alternate serological negatives and positives, corresponding with re-infection episodes.^11,25^

From this perspective, and considering that anti-FCoV antibodies are found in cats with viral RNA both in faeces and tissues of healthy animals and in FIP affected cats,^11,26,27^ the medium-to-high antibody titres recorded in some of the cats of the current study may indicate that these cats had been, or still were, infected with FCoV at the moment of sampling, and therefore it is possible that they were harbouring the virus in tissues. This hypothesis is supported by the finding that some testicles were RT-PCR positive, but always negative at immunohistochemistry. This is not surprising, as PCR is characterised by a higher analytical sensitivity compared with IHC.^28,29^ However, RT-PCR is performed on homogenised samples, thus not allowing us to determine which cellular line of the testicle was infected.

It is important to highlight that only one of the cats with viral RNA in the testicle and with available serum was seronegative, while all the other cats with PCR-positive testicles had titres ranging from 1:100 to 1:400. In the light of what has been discussed above, this may be explained by two hypotheses. The first hypothesis is that the cats were viraemic but with a blood viral load too low to be detected by standard PCR, and the virus was present only in the vessels or in the plasma contained in the testicle. However, the examination with RT-qPCR, which is more sensitive than standard PCR, makes this hypothesis unlikely, as well as the fact that the only viraemic cat, even if with a very low viral load, was PCR negative on testicles.

Another hypothesis, as already demonstrated, is that the examined section for IHC did not include the cells infected by FCoV, which were present in the sections used for RT-PCR instead.^2,3^ In any case, the section used for PCR was carefully handled to avoid haematic contamination as much as possible and therefore it is unlikely that testicles were falsely positive owing to contaminating FCoV genome. Also, the presence of FCoV in the testicular vessels would not explain why the same positivity was not found on blood, from which a larger amount of sample was used for RNA extraction. The most likely hypothesis is that the virus was isolated in the testicular compartment through the blood–testis barrier, as already demonstrated with the blood–brain barrier, thus explaining the discordant results between peripheral blood and testicles.^30^

Interestingly, the only FIP-affected cat had a negative RT-PCR result on the testicle sample. While it was not possible to perform serology and PCR on blood, several tissues from this cat were analysed for diagnostic purposes. All tissues examined were negative on both PCR and IHC, except for brain and cerebellum, which were the only organs harbouring the typical FIP lesions along with intralesional antigen, and a mesenteric lymph node, which was positive on PCR only. This finding supports evidence of a higher analytical sensitivity of RT-PCR and also the fact that positive PCR results do not allow us to distinguish between FIP-affected and FCoV-infected healthy cats.^4,29^ Moreover, the absence of typical histological lesions, as well as of positive IHC, demonstrates that genital involvement is rare during FIP, especially in non-effusive and localised forms, and probably also testicle involvement in FCoV-infected healthy cats.^17,18^

None of the semen samples were RT-PCR and RT-qPCR positive for FCoV. Only in one cat, for which both testicle and semen samples were available, were results discordant, with positive RT-PCR for the testicle sample, but negative for the semen sample. Also, it cannot be excluded that the virus was present on the stromal or vascular tissues of the testicle and not in germinal cells, leading to a negative PCR result on semen. Unfortunately, the negative IHC results, likely due to the low amount of virus as hypothesised above, does not allow us to further elucidate this aspect. It is also important to consider that the diagnostic sensitivity of RT-PCR and RT-qPCR on feline semen is unknown; in this study we applied the method of RNA extraction from semen that is described to have the best analytical sensitivity in comparison with other methods.^31^ Therefore, although unlikely, as this method has been successfully used in other studies, the presence of false-negative results cannot be excluded.^32^ Moreover, most of the cats from which semen was tested were also seronegative or had low antibody titres. Even though seronegative cats cannot be considered free from infection for the already discussed kinetics of both the virus and the antibody responses, it is possible that cats were not viraemic and that the virus was not systemically spread or was localised in some organs at the time of semen collection.^33^ Unfortunately, for the only cat that was RT-qPCR positive on blood, a semen sample was not available.

## Conclusions

Even if positive PCR results on testicles may suggest the venereal route as a potential way of FCoV transmission, FCoV seems not to localise in the semen of tom cats, and so this route seems to be unlikely. Viral RNA found in testicles could not be correlated with viraemic phases, but this finding needs to be confirmed. In the light of these results, AI seems safer than natural mating, eliminating the contact between animals and diminishing the probability of faecal–oral FCoV transmission.

Owing to the limited number of available semen samples and to the fact that samples were obtained almost exclusively from healthy cats, it would be useful to evaluate these data in a FCoV-endemic population to have more chance of detecting viraemic cats, which may possibly harbour FCoV in semen. In addition, the presence of higher antibody titres may allow evaluation of the potential use of serology as an indicator of viral localisation in tissue/semen. Therefore, further studies on a higher number of samples and evaluating differences in the semen and testicles of cats with higher antibody titres or with positive RT-PCR on blood are needed.
